# Waiting Time and Patient Satisfaction in a Subspecialty Eye Hospital Using a Mobile Data Collection Kit: Pre-Post Quality Improvement Intervention

**DOI:** 10.2196/34263

**Published:** 2022-08-09

**Authors:** Mathew Mbwogge, Nicholas Astbury, Henry Ebong Nkumbe, Catey Bunce, Covadonga Bascaran

**Affiliations:** 1 International Center for Eye Health London School of Hygiene & Tropical Medicine London United Kingdom; 2 Africa Eye Foundation Cameroon Yaoundé Cameroon; 3 Research Data & Statistics Unit The Royal Marsden NHS Foundation Trust London United Kingdom

**Keywords:** waiting time, waiting list, patient satisfaction, quality improvement, clinical audit, ophthalmology, patient-centered care

## Abstract

**Background:**

Waiting time can considerably increase the cost to both the clinic and the patient and be a major predictor of the satisfaction of eye care users. Efficient management of waiting time remains as a challenge in hospitals. Waiting time management will become even more crucial in the postpandemic era. A key consideration when improving waiting time is the involvement of eye care users. This study aimed at improving patient waiting time and satisfaction through the use of Plan-Do-Study-Act (PDSA) quality improvement cycles.

**Objective:**

The objectives of this study were to determine the waiting time and patient satisfaction, measure the association between waiting time and patient satisfaction, and determine the effectiveness of the PDSA model in improving waiting time and satisfaction.

**Methods:**

This was a pre-post quality improvement study among patients aged 19 to 80 years, who are consulting with the Magrabi International Council of Ophthalmology Cameroon Eye Institute. We used PDSA cycles to conduct improvement audits of waiting time and satisfaction over 6 weeks. A data collection app known as Open Data Kit (Get ODK Inc) was used for real-time tracking of waiting, service, and idling times at each service point. Participants were also asked whether they were satisfied with the waiting time at the point of exit. Data from 51% (25/49) preintervention participants and 49% (24/49) postintervention participants were analyzed using Stata 14 at .05 significance level. An unpaired 2-tailed *t* test was used to assess the statistical significance of the observed differences in times before and after the intervention. Logistic regression was used to examine the association between satisfaction and waiting time.

**Results:**

In total, 49 participants were recruited with mean age of 49 (SD 15.7) years. The preintervention mean waiting, service, and idling times were 450 (SD 96.6), 112 (SD 47), and 338 (SD 98.1) minutes, respectively. There was no significant association between patient waiting time and satisfaction (odds ratio 1, 95% CI 0.99-1; *P*=.37; *χ*^2^_3_=0.4). The use of PDSA led to 15% (66 minutes/450 minutes) improvement in waiting time (*t*_47_=2; *P*=.05) and nonsignificant increase in patient satisfaction from 32% (8/25) to 33% (8/24; *z*=0.1; *P*=.92).

**Conclusions:**

Use of PDSA led to a borderline statistically significant reduction of 66 minutes in waiting time over 6 weeks and an insignificant improvement in satisfaction, suggesting that quality improvement efforts at the clinic have to be made over a considerable period to be able to produce significant changes. The study provides a good basis for standardizing the cycle (consultation) time at the clinic. We recommend shortening the patient pathway and implementing other measures including a phasic appointment system, automated patient time monitoring, robust ticketing, patient pathway supervision, standard triaging, task shifting, physician consultation planning, patient education, and additional registration staff.

## Introduction

### Background

Long waiting time can significantly increase costs and be a major determinant of the satisfaction of those seeking health care services [1]. Patient experience and satisfaction are closely linked to the quality of care that users attribute to health care [2,3]. Although quality of care does not necessarily translate into patient satisfaction, it can be a major predictor [4]. Patient experience and satisfaction can also be dependent on the time patients spend in clinics during their consultation [5]. The reduction of waiting time has been a key concern, especially for ambulatory hospitals, owing to increasing outpatient demands [6]. Efficient management of patient flow in hospitals ensures high quality of care [7]. It has been reported that patient flow management as part of a hospital quality improvement strategy warrants continuous attention and should involve all staff [7]. Evidence suggests that there is a strong negative correlation between waiting time and patient satisfaction [8,9]. User dissatisfaction has been strongly linked to waiting times, with users spending more time in waiting than being attended to [10]. It is believed that the routine task of health care staff is to perform their work and improve it [11]. However, the ability to reduce waiting time and improve services may be limited by service capacity [12].

In ophthalmology, long waiting time and the dissatisfaction of those seeking eye care have been worsened by the COVID-19 pandemic [13]. Apart from affecting patient satisfaction, system delays also affect health care program delivery [14]. Waiting time has been identified as one of the major challenges in managing workflow in eye hospitals because of the growing number of those in need of eye care [15].

Between 2010 and 2019, the number of people with blindness increased by 10.8% (95% unit interval 8.9%-12.4%) and moderate to severe visual impairment increased by 31.5% (95% unit interval 30%-33.1%) [16]. Sub-Saharan Africa faces severe limitations for well-trained eye care personnel [17]. In Cameroon, it is estimated that 250,000 people are blind and 600,000 are visually impaired. The prevalence of blindness in Cameroon is one of the highest in the world, and there is no government health budget allocation specific to eye health [18].

The concept of waiting time presents different meanings in different contexts. In countries with a regularized appointment system such as the United Kingdom, it is the time spent from booking an appointment to when the person attends the appointment [19]. In low-income economies such as Cameroon, waiting time is the time a patient spends at the clinic to obtain a complete health check [20].

Hospital waiting time is a major concern in Cameroon as in many other countries [21]. The current evidence regarding quality improvement specific to waiting time in hospitals in Cameroon is lacking [22]. The problem of long waiting times in clinics in Cameroon can primarily be attributed to poor management [23], and there is strong evidence that waiting time in Cameroonian hospitals is the main cause of dissatisfaction when accessing health care [24]. Its understanding will help in defining the measures of change needed for its improvement. The problem of long waiting times at the Magrabi International Council of Ophthalmology Cameroon Eye Institute (MICEI) escalated owing to the increase in patient volume. Conscious of the need to deliver high-quality eye care services, the eye institute capped its daily patient visits, in part, to deal with the overwhelming number of patient complaints about waiting time. Following this, MICEI management sought to investigate the time that patients spend at the clinic and propose measures of improvement.

### Study Rationale

Our choice of Cameroon stems from the fact that apart from the lack of any previous study that primarily sought to improve waiting time and satisfaction in Cameroon, waiting time was found to be the main reason for patient complaints at the newly established eye hospital (MICEI) in Cameroon. The study was the first of its kind that was specific to ophthalmology in Cameroon. However, we found quality improvement interventions undertaken in other health areas [22,25,26]. One sought to improve waiting time by means of hospital-wide quality improvement, using the Strengthening Laboratory Management Toward Accreditation model [22]; another sought to improve early infant diagnoses coverage, timely return of HIV test results, and initiation of antiretroviral treatment using the Quality Improvement Collaborative approach [25]; and another sought to improve the adherence and cure of patients with tuberculosis, by using SMS text message reminders [26]. We also found 2 studies [23,24] that aimed at investigating patients’ satisfaction with the quality of health services [23] and the undertaking of antiretroviral treatment [24].

This study was based on the model of Plan-Do-Study-Act (PDSA) [27]. This 4-stage model was proposed by Deming as a simple way to undertake quality improvement interventions in health care. It involves making continuous cyclical improvements geared toward achieving what works best for care users.

The use of pre-post quasi-experimental designs [28,29] and the PDSA model in health care [30] in general and in ophthalmology [31] in particular, has been widely reported. A similar study was conducted in Ethiopia using an appointment system [20]. In addition, there is evidence of use of the Open Data Kit (ODK) developed by Get ODK Inc, in health care projects in Cameroon [32].

### Specific Objectives

This study had three objectives: (1) determine the waiting time and satisfaction, (2) measure the association between waiting time and satisfaction, and (3) measure the effectiveness of the PDSA model in improving waiting times and satisfaction.

## Methods

### Study Setting

This study was conducted at the MICEI from June 15, 2018, to July 28, 2018. MICEI is a subspecialty eye hospital and training center, with an average of 300 daily outpatient visits [33]. The center is the only tertiary eye institute in Cameroon, with 72-bed capacity, 8 ophthalmologists, 8 ophthalmic nurses, and approximately 70 full-time staff.

### Contextual Factors

Study feasibility was carefully examined by assessing some contextual factors that are likely to affect success [34]. The study was made context-specific by using the Model for Understanding Success in Quality [35]. We calculated the Model for Understanding Success in Quality score using an Excel template developed by the East London National Health Service Foundation Trust [36], as shown in Table S1 in Multimedia Appendix 1.

The eye care center is suburban, 25 km away from the city center of the country’s capital. The Center Region is host to 8 other eye clinics delivering general ophthalmology services in public and private hospitals. Enabling factors include motivated executive toward quality improvement, well-structured microsystem with state-of-the-art equipment, the institute’s aim to become a center of excellence, and high donor expectations. In addition, MICEI runs a patient-based and tiered pricing model similar to that of the Aravind Eye Care System in India, which is different from the disintegrated hospital-based eye care delivery within Cameroon. Other positive factors were the availability of stationery and printing of study materials at the hospital and the hospitality of the staff.

### PDSA—Plan and Do Phase: Intervention

#### Overview

**Figure 1:**
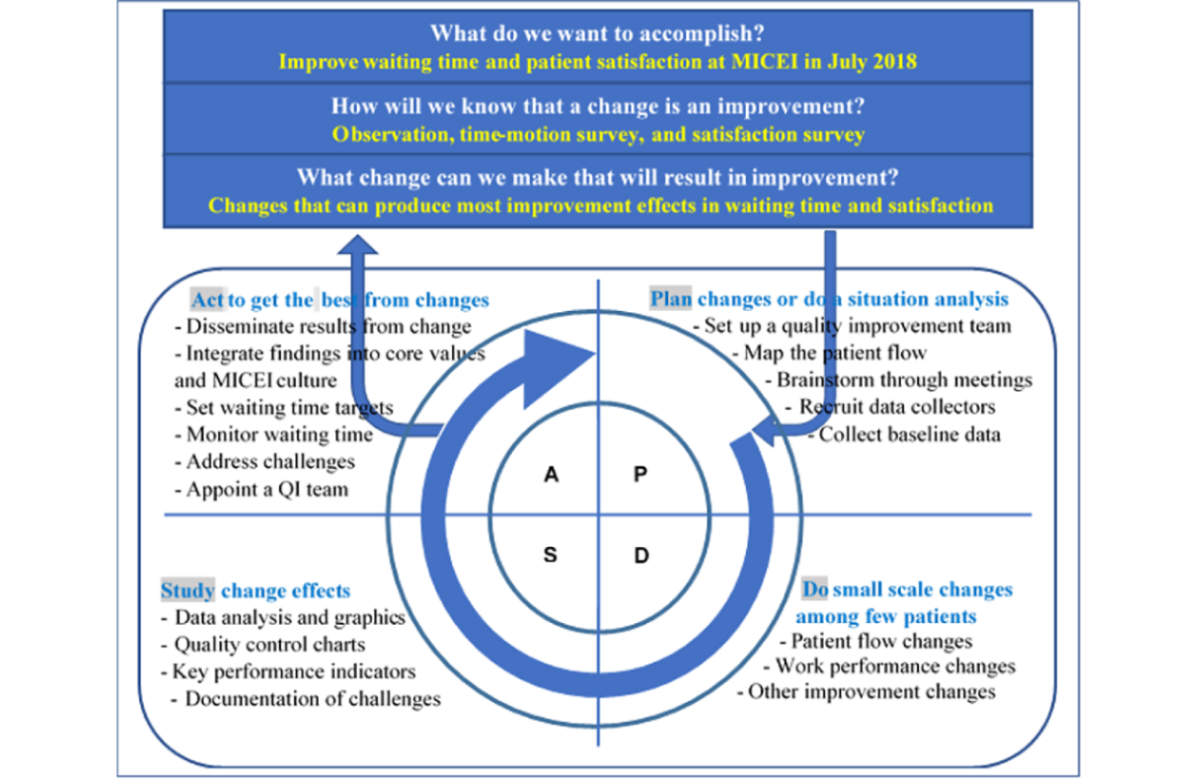
Plan-Do-Study-Act conceptual framework. Licensed under the Open Government License. MICEI: Magrabi International Council of Ophthalmology Cameroon Eye Institute; QI: quality improvement.

This was a 2-step person-centered quality improvement intervention using the PDSA model. The first step involved situation analysis of the waiting time and mapping of the patient flow. On the basis of this analysis, best-fit measures were introduced to offset delays in the waiting time. Figure 1 shows an adapted PDSA conceptual framework of the intervention [37].

#### Recruitment of Participants

Study participants were recruited from patients consulting with the MICEI between June 2018 and July 2018, using nonprobabilistic sampling [38]. Participants were randomly approached at the point of entry by 2 trained data collectors and introduced to the study if they met the inclusion criteria, and only those who voluntarily consented were enrolled. The inclusion criteria were the following: aged between 18 to 80 years, seeking ophthalmic consultation, and able to understand and speak either English or French. The exclusion criteria were the following: incapacity to provide consent; surgical and postoperative appointments; and patients not following the normal flow, such as those in the *fast track* and *very important person* categories.

#### Data Management

Data collection was performed using ODK [39]. A data form was built using Microsoft Excel 2010 and validated using the web-based Microsoft Excel Spreadsheet Form (XLSForm Online version 1.2.0). Then, the Excel data form was converted to a version (XML) compatible with the server (ODK back end) using the downloadable Microsoft Excel Spreadsheet Form (ODK-XLSForm Offline version 1.6.0; Multimedia Appendix 2) and uploaded to ODK Aggregate server (open-source Java server) with a personalized user ID and password. As this study was conducted in a predominantly French-speaking region, all data forms and patient information materials were translated into French to suit participants.

Huawei MediaPad T2 10.0 Pro and Samsung Galaxy Note 10.1 android tablets with installed ODK Collect application (open source) for requesting data forms from the server were used to collect real-time data on waiting time and patient satisfaction.

To check for completeness, 2 dry runs were performed and the data form was modified before the start of the intervention. Data collection was automated, thereby reducing errors. The data form was built such that each question must be answered before proceeding to the next. All the filled data forms were verified by the principal investigator for completeness before submitting to the server.

The latest version of ODK Briefcase downloaded and installed on Windows 10 was used to extract the data set from the Aggregate server. Then, this was exported as a CSV file and loaded into Stata 14 for analysis.

#### Quality Improvement Team

A quality improvement team was set up, including the principal investigator, pediatric ophthalmologist, medical records officer, senior outpatient nurse, head of investigations, nurse assistant, optical technician, and facility manager. The team met once every week on less busy days from 7 to 8 AM, to provide feedback on daily challenges and propose solutions. The team aimed to reduce waiting time by 25%.

#### Data Collectors

A total of two data collectors (an advanced-level holder and a university student) purposely recruited for the study were trained using a standard operating procedures manual developed for the study.

#### Dry Run and Testing

After 2-day training, a dry run was performed on 2 consenting patient volunteers. On the basis of the challenges, the data form was modified to account for interunit counterreferrals (owing to back-and-forth movements) and include the option, *other*, to some of the questions to make answers more flexible. The questionnaire was finalized after a second dry run, converted, and resubmitted to the server.

#### Changes Proposed

The patient flow was mapped, and all consultation rooms were identified according to room numbers. Patient flow bottlenecks were identified through brainstorming and direct observations. On the basis of an interim analysis of data collected from 51% (25/49) of the participants, the following measures were proposed to potentially reduce waiting time:

1. A time monitor sheet to record the start and finish times at each service point.
2. Introduce a second receptionist for the separate handling of reviews.
3. Introduce a numbering system for all patients (reviews and new patients alike).
4. Regularly supervise the patient flow for on-the-spot handling of bottlenecks.
5. Appoint an experienced ophthalmic nurse for effective triaging of patient files.
6. Educate patients on patient flow, for orientation and reduction of turnaround time.
7. Standardize waiting time by defining the duration for a full consultation.
8. A phasic appointment system that includes associating a nurse assistant to each ophthalmologist, to take notes and book appointments, and the proactive sorting of patient files a day before the booked appointments. Each day is divided into slots corresponding to the maximum number of patients a physician is able to handle.
9. Grant ophthalmic nurses’ permission to discharge less complicated cases.
10. Color zoning of the general ophthalmology department to know who is waiting for whom.

### PDSA: Study of the Intervention Phase

#### Approach to Impact Assessment

A PDSA-led pre-post quasi-experimental design was used to measure the effectiveness of the intervention, from June 15, 2018, to July 28, 2018. This method was particularly important because we wanted to address two key aspects of quality: clinical effectiveness through waiting time and patient experience through patient satisfaction [40]. We used the before-after design [29] to keep the intervention as close to reality as possible. Moreover, it was not ethical to conduct a pre-post study with a control group as this was a single-center study [29]. In addition, evidence on the use of PDSA in quality improvement interventions has been well documented [41-43]

#### Attributing Results to the Intervention

A total of 49 participants from randomly arriving patients at the eye institute were invited to participate in a time-motion and satisfaction survey at 2 time points (n=25, 51% participants before the intervention and n=24, 49% participants after the intervention). Data collectors randomly approached participants at the point of entry, explained the study to them, and enrolled only those who provided voluntary consent. Through a process of shadowing, data collectors recorded the time spent at each service point, from entry to exit. At the exit, patients were asked whether they were satisfied and the reasons for their dissatisfaction, if relevant. We determined that the results were owing to the intervention by assessing and comparing the waiting time and patient satisfaction of the 2 samples.

### Measures

#### Processes and Outcomes

The duration of a full consultation day was investigated using waiting time as the primary outcome variable. Waiting time was defined as the time spent in the microsystem, from entry to exit [20]. It was a continuous variable made up of (1) service time, which is the time the patient is being served and in contact with staff, and (2) idling time, which is the time the patient spends between service points, waiting to be served. The secondary outcome variable was patient satisfaction, defined as the patient-reported satisfaction with waiting time and service. This was used to determine whether waiting time was a good determinant of patients’ satisfaction. Other variables included participants’ sociodemographic variables.

#### Assessment of Contextual Factors

Direct observations, quality improvement meeting sessions, and interim analysis, including the use of data visualization techniques (scatter and box plots), were used to determine any unusual data points that can be attributed to contextual factors. Abnormal data points were identified by calculating the lower (Q1–1.5[Q3–Q1]) and upper (Q3+1.5[Q3–Q1]) fences. Data points that fell outside these limits were investigated further.

### Data Analysis

#### Waiting Time and Satisfaction

All statistical analyses were performed using Stata 14 at .05 significance level. On the basis of our sample size, the Shapiro-Wilk test for the pretest sample (*z*=1; *P*=.10) and the posttest sample (*z*=−0.98; *P*=.80) showed that both samples were assumed to be drawn from a normal distribution [44]. In addition, the skewness and kurtosis tests for the first sample (skewness: *P*=.30; kurtosis: *P*=.90) and the second sample (skewness: *P*=.50; kurtosis: *P*=.80) fulfilled the normality hypothesis. On the basis of these tests, we used the parametric approach for our data analysis. The mean waiting, service, and idling times were calculated. Patients’ satisfaction was analyzed using frequencies. Box plots were used to compare waiting times between men and women according to type of patient. A difference in means plot was also used to visually inspect and compare the means between categorical variables including gender, age group, arrivals, diagnosis, and residence.

#### Association of Waiting Time and Satisfaction

Logistic regression [45] with reported odds ratios (ORs) was performed to establish the existence of any association between waiting time and patient satisfaction. Participants’ satisfaction was modeled with waiting time, age, and gender using the logistic regression, and ORs with 95% CIs were calculated.

#### Effectiveness of PDSA

Independent sample 2-tailed *t* test [46] was used to compare the waiting time and satisfaction of the preintervention and postintervention groups. Box plots and pie charts were used to visually examine the pre-post intervention effects on waiting time and patient satisfaction, respectively, according to gender and type of patient.

### Ethics Approval

Consistent with the Helsinki Declaration of 1975, the protocol for this study was developed and approved by the ethics committee of the London School of Hygiene and Tropical Medicine (15444). Ethics approval was also obtained from the institutional review board of MICEI (0003/L/DG/DM/PA/KBG). All the participants provided written informed consent. All the data forms submitted to the server were encrypted using a pair of public keys. Participants received reimbursement for their consultation fees.

## Results

The study findings are reported in accordance with the revised Standards for Quality Improvement Reporting Excellence (version 2.0) guidelines [47].

### Participant Demographics

A total of 49 participants, 15 (31%) of whom were reviews, participated in the study. Their mean age was 49 (SD 15.7) years, ranging from 19 to 80 years (25/49, 51% were women). Participants were recruited into two consecutive samples (preintervention sample and postintervention sample) and matched for age and self-reported sex. The mean age for the preintervention arm (25/49, 51% of the participants; 13/25, 52% were women) was 49.3 (SD 14.6) years and that for the postintervention arm (24/49, 49% of the participants; 12/24, 50% were women) was 49.6 (SD 17) years. Most patients (38/49, 78%) arrived between 6 and 9 AM for their consultation. Table 1 presents the sociodemographic characteristics of the study participants.

**Table 1:** Sociodemographic characteristics (N=49).

Characteristics		Preintervention participants (n=25), n (%)	Postintervention participants (n=24), n (%)
**Age (years)**			
	15-24	1 (4)	3 (13)
	25-54	14 (56)	10 (42)
	55-64	5 (20)	7 (29)
	65-80	5 (20)	4 (17)
**Gender^a^**			
	Men	12 (48)	12 (50)
	Women	13 (52)	12 (50)
**Patient type**			
	New	18 (72)	16 (67)
	Review	7 (28)	8 (33)
**Marital status**			
	Married and cohabiting	17 (68)	14 (58)
	Single	6 (24)	7 (29)
	Divorced and widow	2 (8)	3 (13)
**Residence**			
	Littoral	0 (0)	2 (8)
	Far North	1 (4)	1 (4)
	Center	20 (80)	20 (83)
	West	2 (8)	0 (0)
	Northwest	0 (0)	1 (4)
	South	1 (4)	0 (0)
**Origin**			
	Littoral	0 (0)	1 (4)
	Center	14 (56)	8 (33)
	West	9 (36)	14 (58)
	Northwest	1 (4)	0 (0)
	North	0 (0)	1 (4)
	South	1 (4)	0 (0)
**Work status**			
	Formal	9 (36)	6 (25)
	Informal	8 (32)	13 (54)
	Others	8 (32)	5 (21)
**Education**			
	None	1 (4)	1 (4)
	Elementary	3 (12)	1 (4)
	GCE^b^—ordinary level	6 (24)	11 (46)
	GCE—advance level	3 (12)	2 (8)
	University	4 (16)	7 (29)
	Doctorate	8 (32)	2 (8)
**Travel time**			
	<1 hour	18 (72)	19 (79)
	A few hours	5 (20)	4 (17)
	Half a day	1 (4)	0 (0)
	1-2 days	1 (4)	1 (4)
**Transport means**			
	Private	6 (24)	6 (25)
	Public	18 (72)	18 (75)
	Motorbike	1 (4)	0 (0)
**Arrival time**			
	5-7 AM	14 (56)	5 (21)
	7-9 AM	7 (28)	14 (58)
	9-11 AM	4 (16)	5 (21)

^a^Self-reported.

^b^GCE: General Certificate of Education.

### Patient Pathway (Patient Flow)

**Figure 2:**
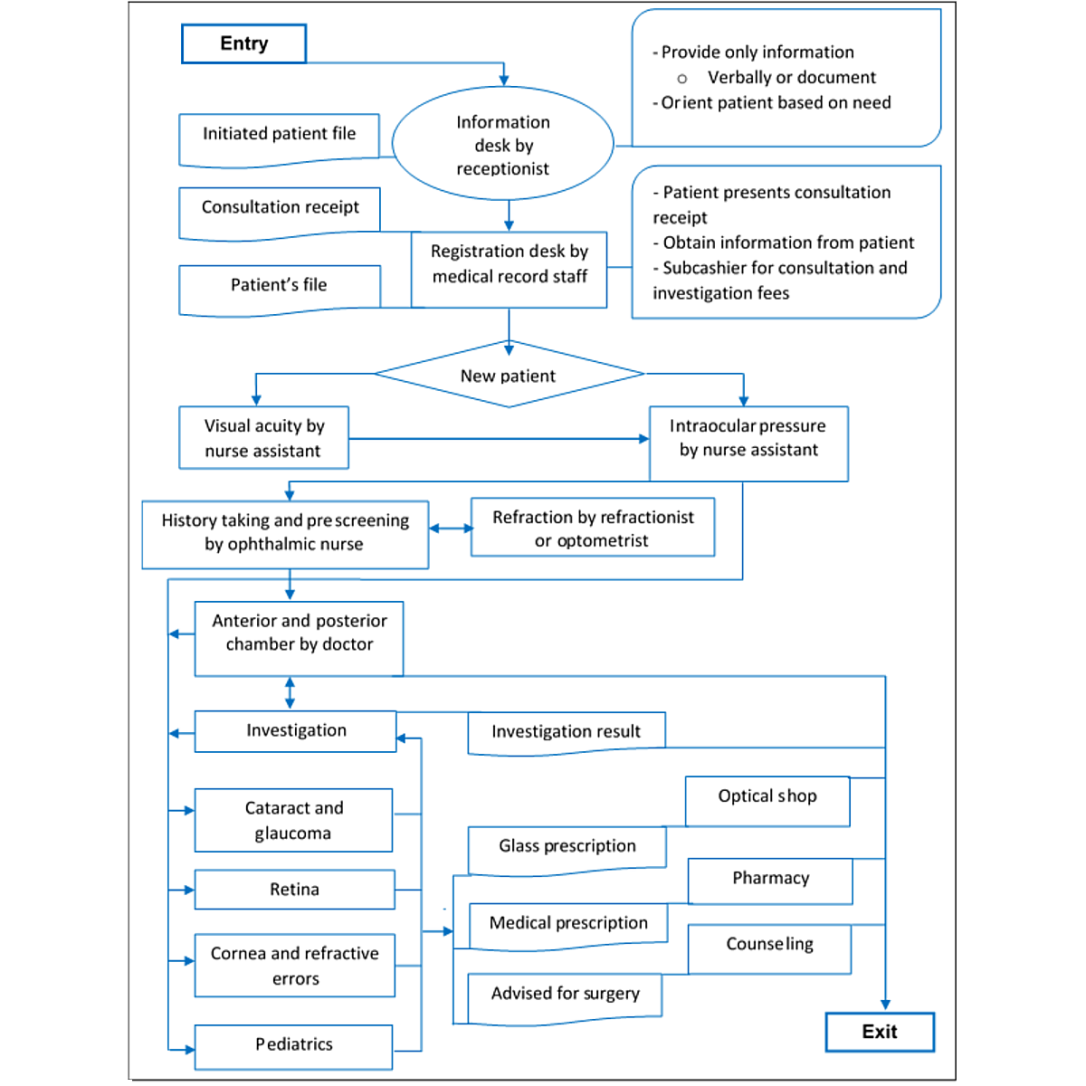
Authors’ conception—patient pathway protocol.

The patient flow chart starts at the gate where patients are handed a number upon arrival. Medical record files are initiated at the reception by calling the patients based on numbers. Patients are also advised on the consultation fee based on the consultation option chosen (very important person, fast track, or standard). Patients are registered in the medical records upon presentation of a cash receipt of the consultation fee. If patients are on a repeat visit, their medical record file will have to be retrieved by the medical records officer to proceed to the next service point. In the general ophthalmology unit, visual acuity, blood pressure, and intraocular pressure are measured by assistant ophthalmic nurses. The visual acuity determines whether patients should be refracted. Patients are prescreened by an ophthalmic nurse with the help of a slit lamp before seeing the general ophthalmologist. The general (outpatient) ophthalmologist may request for mydriatic eye drops to be instilled if necessary. Then, he refers patients to subspecialty units based on the anterior and posterior chamber assessments (using a slit lamp). The flow is such that there may be back-and-forth movements owing to counterreferrals. At the end of the intervention, 96% (47/49) of the participants had visited the general ophthalmology department. Altogether, 49% (24/49) of the participants had visited the cataract and glaucoma unit and 31% (15/49) had visited the cornea and refractive errors unit. There was no marked difference in service point visits according to gender and sample. Figure 2 shows the patient flow at the clinic.

### Intervention Time Line

In the preintervention phase, 51% (25/49) of the participants participated in a time-motion and satisfaction survey. On the basis of the interim analysis, changes were implemented. The second group of 49% (24/49) participants was recruited for the time-motion survey after the changes, and the 2 groups were compared.

The first 7 changes were implemented, which includes the following: (1) a time monitor sheet to record the start and finish times at each service point, (2) introduction of a second receptionist for the separate handling of review patients, (3) expansion of the numbering system to include all patients, (4) patient flow supervision for on-the-spot handling of bottlenecks, (5) triaging of patient files led by assistant nurses at the general ophthalmology department, (6) proactive sorting of patient files in the medical records, and (7) regular patient education by a medical record staff. These changes were implemented simultaneously as a package.

Of the 10 originally proposed changes, three changes (ie, standardization of waiting time by defining the duration for a full consultation, granting ophthalmic nurses the permission to discharge less complicated cases, and color zoning of the general ophthalmology department) could not be implemented owing to cost and time constraints. For instance, color zoning of the outpatient waiting area required a formal contract award procedure. Other three measures, including the phasic appointment system, effective triaging, and patient education, could not be fully implemented owing to staff shortage, lack of qualified nurses, and lack of audiovisual materials, respectively.

**Figure 3:**
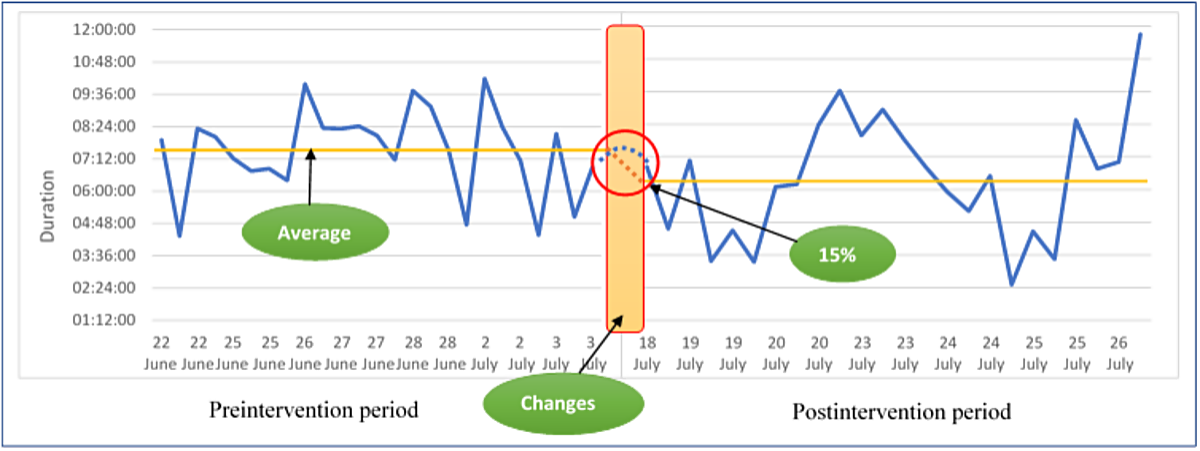
Time line of time-motion and satisfaction survey.

Figure 3 displays the waiting time series with the intervention effect.

### Waiting Time and Patient Satisfaction

At baseline, the mean waiting time (service time and idle time) for a comprehensive eye examination at the MICEI was found to be 450 (SD 96.6) minutes. The mean service time was 112 (SD 47) minutes and the mean idling time was 338 (SD 98.1) minutes. The idle time (338 minutes) spent by patients was 3 times more than that being served. The service points with high mean waiting times at baseline included room 15 with 204 (SD 86.1) minutes, room 20 with 203 (SD 141.4) minutes, room 13 with 185 (SD 46.1) minutes, room 18 with 161 (SD 63.5) minutes, and room 16 with 99 (SD 97.4) minutes. At baseline, the highest proportion of idling time was among patients going through room 15 (196 minutes/204 minutes, 96%), room 20 (192 minutes/203 minutes, 95%), room 13 (167 minutes/185 minutes, 90%), room 18 (140 minutes/161 minutes, 87%), and room 16 (84 minutes/99 minutes, 85%).

The mean waiting time for men was 472 (SD 86.5) minutes and that for women was 429 (SD 104.1) minutes. Men spent 77% (362 minutes/472 minutes) of the time in idling, whereas women spent 73% (315 minutes/429 minutes) of the time. Table S1 in Multimedia Appendix 3 shows the detailed waiting, service, and idling times by service point and gender.

**Figure 4:**
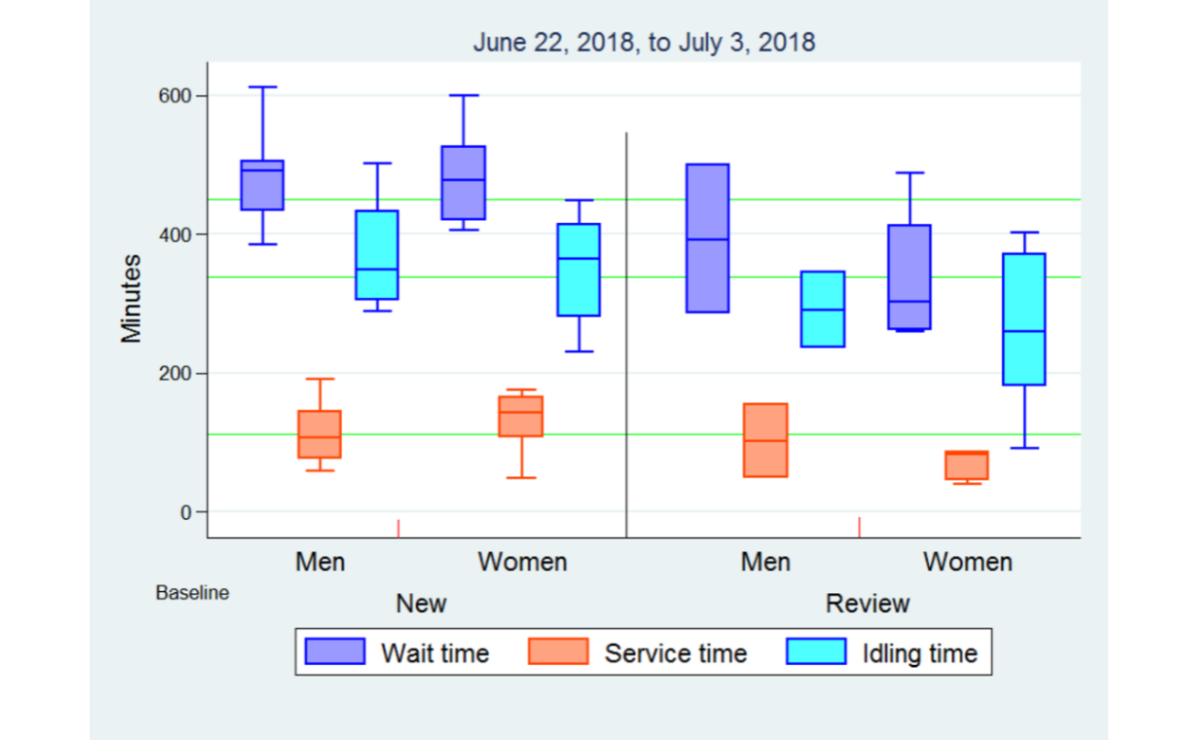
Baseline waiting time of new and review patients by gender.

The mean waiting time for new patients was 485 (SD 67) minutes and that for reviews was 359 (SD 105.8) minutes. Both new patients and reviews spent 75% of the waiting time in idling (364 minutes/485 minutes and 269 minutes/359 minutes, respectively). Figure 4 shows the baseline waiting, service, and idling times of new and review patients by gender.

Of the 51% (25/49) of the participants who participated in the baseline survey, 32% (8/25) reported that they were satisfied with the waiting time, 63% (5/8) of whom were women and 75% (6/8) were new patients. Among the participants who reported to be dissatisfied (17/25, 68%), 76% (13/17) complained of long waiting time as the main reason for dissatisfaction, whereas 24% (4/17) complained of queue jumping.

At baseline, 28% (7/25) of the participants in the preintervention sample was reviews. All 7 participants reported that they were dissatisfied with their first visit to the clinic. Of these participants, 43% (3/7) agreed that they were satisfied with the current visit.

### Association of Waiting Time and Satisfaction

We performed binary outcome logistic regression because satisfaction was a binary outcome. Waiting time was not a good predictor of satisfaction, as the negative association (*z*=−0.9) was not statistically significant (OR 1, 95% CI 0.99-1; *P*=.37; *χ*^2^_3_=0.4). Further investigation by gender and age group did not show any significant difference.

### Effectiveness of PDSA

An independent sample *t* test showed that the mean waiting time reduction from 450 (95% CI 409.7-489.5) minutes at baseline to 384 (95% CI 327.8-440.6) minutes after intervention was not statistically significant, with 15% (66 minutes/450 minutes) reduction in mean waiting time (*t*_47_=2; *P*=.05). The mean service time significantly reduced from 112 (95% CI 92.5-131.3) minutes to 85 (95% CI 71.9-98) minutes (*t*_47_=2.4; *P*=.02), whereas the mean idling time reduced from 338 (95% CI 297.2-378.2) minutes to 299 (95% CI 248.3-350.3) minutes. The reduction in waiting time was mainly driven by high service rate, as the difference of 38 (95% CI −24.7 to 101.5) minutes in idling time was not statistically significant (*t*_47_=1.2; *P*=.20). Tables S1 and S2 in Multimedia Appendix 3 show the effects of the intervention on waiting and service times. The mean waiting time for women increased by 2% (10 minutes/429 minutes), whereas that for men reduced by 30% (142 minutes/472 minutes). Service time for men was 1.6 times (33 minutes/20 minutes) more likely to reduce than that for women. In addition, the idling time for men was similar before (362 minutes/472 minutes, 77%) and after the intervention (253 minutes/330 minutes, 77%), whereas that for women increased from 73% (315 minutes/429 minutes) to 79% (345 minutes/438 minutes). A detailed distribution of waiting time is provided in Table S1 in Multimedia Appendix 3.

**Figure 5:**
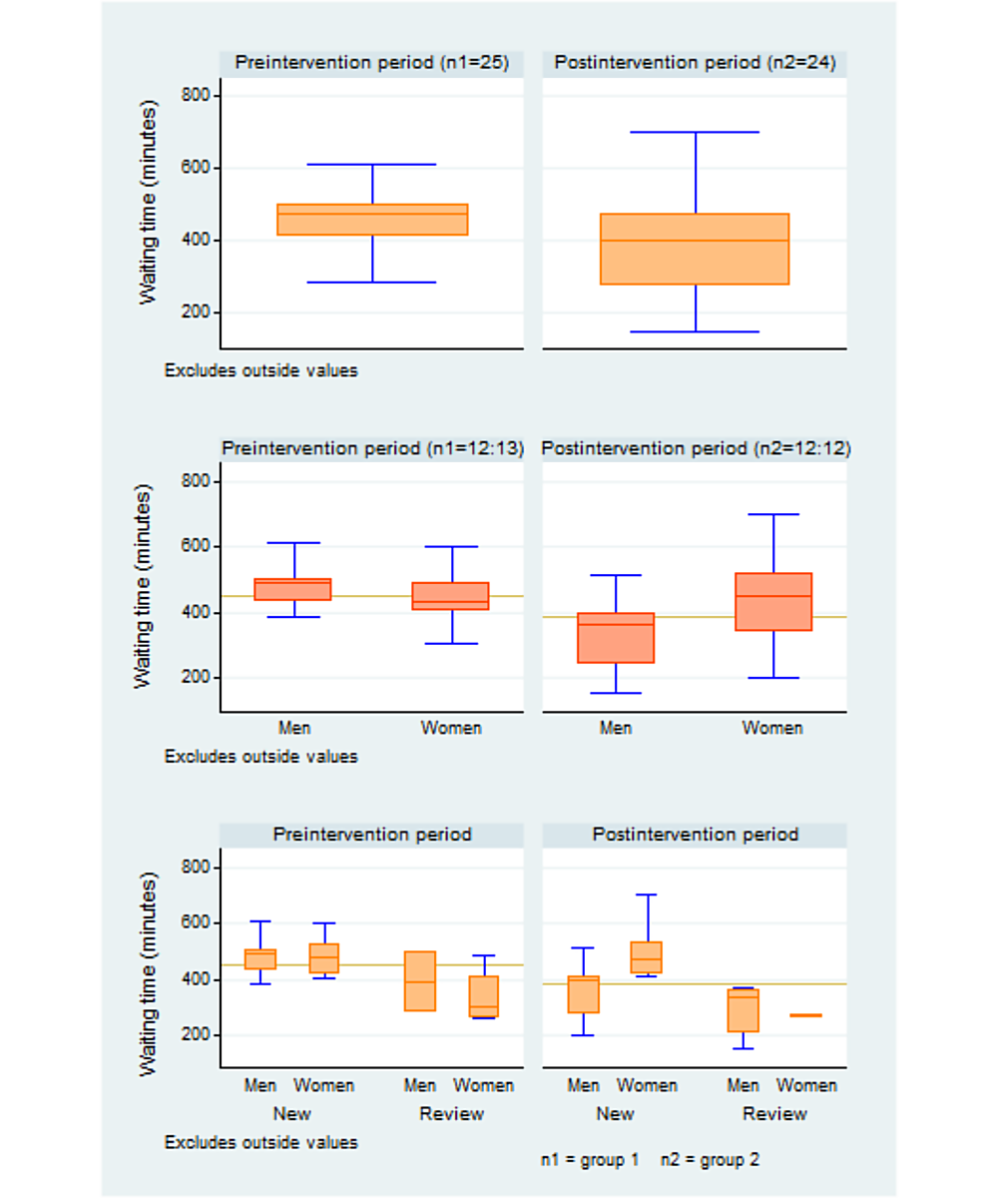
Intervention effect on waiting time.

The mean waiting time for new patients reduced by 11% (53 minutes/485 minutes) and that for reviews reduced by 20% (71 minutes/359 minutes). The intervention was approximately twice as likely to have a positive impact on the waiting time of reviews. Figure 5 shows the intervention’s effect on waiting time.

**Figure 6:**
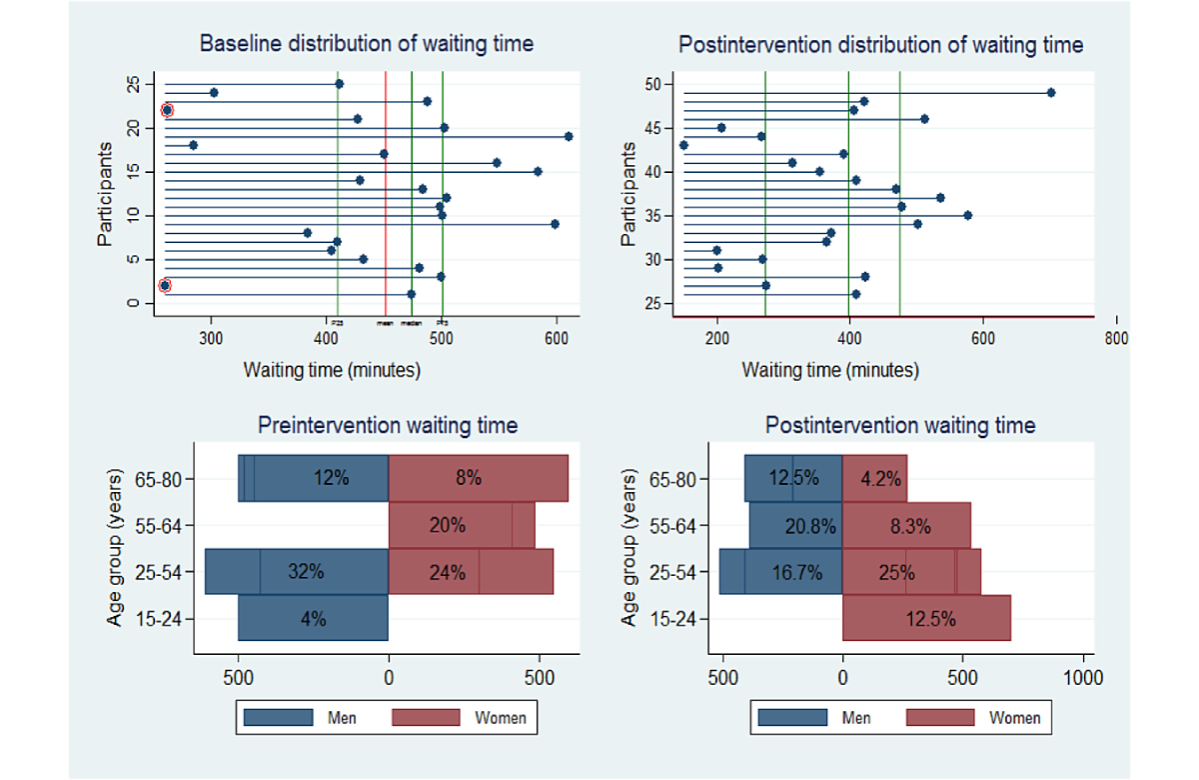
Comparison of preintervention and postintervention waiting time.

Figure 6 shows an overview of the intervention’s effect on the distribution of waiting time and satisfaction.

**Figure 7:**
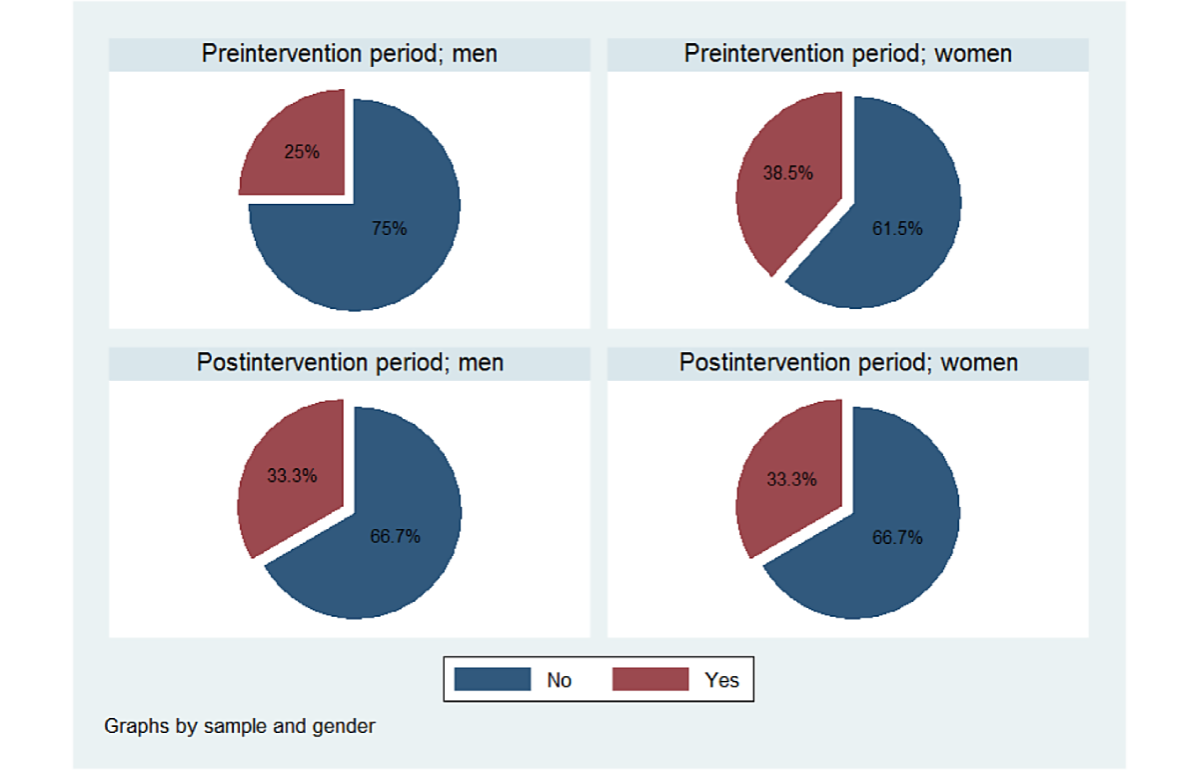
Pre-post comparison of patient satisfaction.

The satisfaction with waiting time increased slightly from 32% (8/25) at baseline to 33% (8/24) after the intervention. This difference (0.01, 95% CI −0.2 to 0.3) was not statistically significant (*z*=0.1; *P*=.9). The percentage of new patients who reported to be satisfied increased from 33% (6/18) to 38% (6/16), whereas that for reviews decreased from 29% (2/7) to 25% (2/8). In addition, those who said that they were satisfied tended to be older than those who did not. Figure 7 shows the satisfaction with waiting time by gender.

### Unintended Outcomes

**Figure 8:**
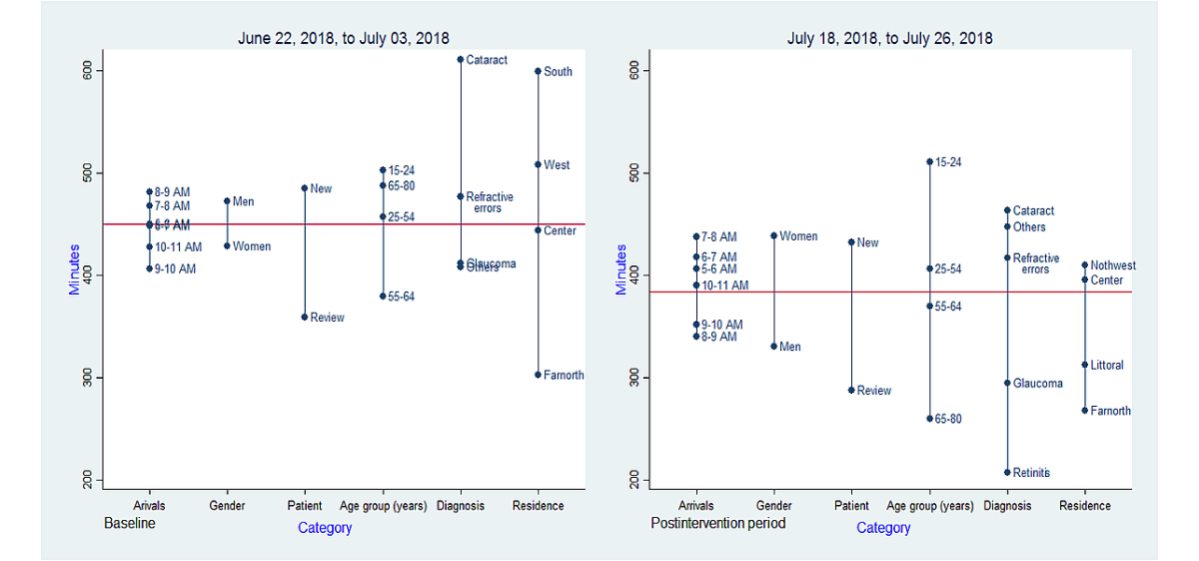
Difference in means by category.

The intervention led to unexpected increase in the waiting time for the general ophthalmologist examination. In addition, the intervention appeared to have affected women adversely, as evidenced by the slight increase reported in the waiting time. A mean comparison across variables showed that this effect was more marked for women in the age group of 15 to 24 years (Figure 8).

Further investigation showed that the 6% (3/49) of women who belonged to the age group of 15 to 24 years were enrolled after the intervention, thus giving a wrong indication of an adverse effect.

## Discussion

### Principal Findings

We found mean waiting time of 450 (SD 96.6) minutes, mean service time of 112 (SD 47) minutes, and mean idling time of 338 (SD 98.1) minutes. The PDSA intervention led to 15% (66 minutes/450 minutes) improvement in mean waiting time (*t*_47_=2; *P*=.05), from 450 (95% CI 409.7-489.5) minutes at baseline to 384 (95% CI 327.8-440.6) minutes after the intervention. Only one-third of the participants reported being satisfied with the waiting time (8/25, 32%) at baseline. Waiting time was not found to be associated with satisfaction (OR 1, 95% CI 0.99-1; *P*=.37; *χ*^2^_3_=0.4).

### Comparison With Previous Studies

#### Baseline Waiting Time

Other studies have reported high mean clinic waiting times; however, we have not found any reports as high as our finding. Mean waiting time of 274 (SD 103.4) minutes was reported among adults visiting the University of Port Harcourt Teaching Hospital in Nigeria, which was lower than the 450 (SD 96.6) minutes we found in this study [48]. The sample size (n=401) was much larger than ours, and the medical services and patient flow involved significantly few steps for the patient to navigate. The short patient pathway may explain the short time spent at the clinic. The mean waiting time of 104.1 (SD 96.4) minutes found in a study conducted at the Thong Nhat Hospital in Vietnam was also lower than our finding [49]. In that study, patients saw the consultant immediately after registering, and the consultant either recommends a blood or imaging test. The patient revisits the physician and, then, is sent to the pharmacy. This pathway with 5 service points and rapid access to the senior physician can explain the lower waiting time compared with our study, where patients had to visit 12 service points on average. Another study conducted in a teaching hospital in Nigeria reported 160.2 (SD 62.4) minutes of waiting time [50]. In that study, waiting time was defined as the time from registration to seeing the physician, rather than the total visit time that we used in our study. Similarly, a study at the Kintampo Municipal Hospital in Ghana reported a mean total visit time of 303.6 (SD 94.8) minutes (5.06 hours) [51]. Their patient pathway comprised only 6 service points. A pilot quality improvement using PDSA cycles in an operating theater unit of a tertiary hospital in India found the average waiting time at baseline to be 221 minutes [42]. Differences in waiting time measurement can explain the low waiting time, which was limited to time at the operating theater. Similarly, at the Medunsa Oral Clinic in South Africa, the mean total time spent at the clinic among 149 patients was reported to be 235.79 (SD 78.79) minutes, which is approximately 2 times lower than the 450 (SD 96.6) minutes reported in our study [52]. The patient pathway for the dental clinic was simple with only five service points (check-in, reception, diagnostic room, treatment, and checkout). Another study at the Jos University Teaching Hospital in Nigeria showed that the total mean outpatient time was 248 minutes [53]. Again, patients in this study followed a simple patient pathway, being sent to see the physician after registration, after which they were sent to the pharmacy. A waiting time audit among 316 women attending an antenatal clinic in Ghana showed that the mean time spent at the clinic was 6.5 hours, which is close to the 7.5 hours reported in our study [54]. Although the definition of waiting time in their study was similar to that used in ours, the 6.5 hours waiting time in their study was based on the reported time spent at the clinic rather than a time audit, as was the case in our study. As such, no details about the patient pathway were given, but 73% of the participants (n=204) noted that most of the time was spent in waiting to see the physician.

From these findings, it appears that streamlining the patient pathway by reducing the number of service points that the patients have to navigate and giving the patient access to the physician faster may be a good strategy to reduce overall waiting times.

#### Service and Idling Times

In our study, the proportion of idling time increased from 75% (338 minutes/450 minutes) to 78% (299 minutes/384 minutes) after the intervention, even though there was a general reduction in mean waiting time.

Similar studies in other settings also reported high idling times, such as a study in China, among 49 outpatients in an endocrinology center, that reported the idling time to be 89% (150.5 minutes/168.3 minutes) [10]. A multicenter study across 9 clinics in KwaZulu-Natal (South Africa) with a sample size of 1763 (baseline: n=860 and follow-up: n=903) used a health service strengthening framework over 12 months and reported the proportion of idling time after the intervention to be 94% (115 minutes/122 minutes) [55]. Akinyinka et al [56] found the eye clinic service time at a primary care center in Lagos to be 8.2 (SD 2) minutes, similar to our findings of 8.5 (SD 8.8) minutes for the general ophthalmologist in our study. In Southwestern Ethiopia, a study including 853 patients showed that patients spent a total time of 553.4 minutes in going through all service points, of which 50% (274.9 minutes/553.4 minutes) was spent in waiting for services [57]. At the University of Benin Teaching Hospital in Nigeria, the proportion of time spent before seeing the physician was reported to be 85% (22 minutes/146 minutes) [58].

In New York, patients spent 58% (53 minutes/91.9 minutes) of the mean total visit time in waiting to be called into a room (20.1 minutes), for the provider (18.6 minutes), and for the preceptor (14.3 minutes) [59]. Visit time was based on appointment visits, with a much simple patient pathway including only registration and examination room. A study including 555 patients attending a teaching clinic in Sacramento (the United States) reported the time spent at the clinic to be 80.5 (SD 30) minutes, of which 19 (SD 16) minutes were spent idling [60]. Their waiting time was based on an individual appointment system and involved a 2-stage consultation (registration and examination room). Our study was based on a block appointment system with multiple provider service points. A study in a pediatric clinic in the United States reported the idling time to be 20.9 to 23.9 minutes for consultations and 15.8 to 20.32 minutes for the filling of prescriptions, using a Lean Six Sigma model [61]. Their idling times were not computed for the entire patient pathway, as these were the times patients waited before being attended to after registration and the time between paying the prescription bill and being called at the pharmacy, respectively.

This evidence suggests that patients attending clinics in low-income and middle-income settings, in particular, may be spending most of their time in waiting to receive a service, referred to as idling waiting time in our study. It would be pertinent to consider interventions that focus specifically on decreasing the time patients spend between service points and possibly reducing the number of service points in the patients’ pathway. Our intervention decreased the overall waiting time, but likely through a proportionally large reduction in service time rather than idling time. The length of consultation may affect patient safety and clinical effectiveness, and caution should be exercised when introducing measures that reduce the time of consultations, which are already brief [62-66].

#### Reduction in Waiting Time

Our study reports a reduction of 15% in waiting time through the intervention, which is less than the original target of 25% reduction. This can be a result of not being able to implement all the originally planned components of the intervention and the short time between intervention implementation and analysis. In addition, the involvement of physicians in training on the last day of our study led to an unusually high waiting time of 702 minutes for the last participant, thereby affecting our mean results.

Several studies of interventions to reduce waiting times report reductions in the same range as that reported in our study. Racine et al [59] conducted a before-after study, including 844 patients (group 1=426; group 2=418) at a pediatric clinic in the East Bronx in New York and reported a reduction of 15% (13.6 minutes/91.9 minutes) in mean total visit time. The reduction in mean waiting time achieved in our study was also comparable with the 13% (28 minutes/208 minutes) reported in a before-after study using the Lean Six Sigma model, with the National Heart Institute in Cairo, Egypt, over 16 months [67]. In the United States, Ciulla et al [68] achieved 18% reduction through their intervention, using the Lean Six Sigma model. Another study conducted in an emergency department in Singapore over 6 months showed 12% reduction using a similar model [69]. Improvements at the Fujiang Provincial Hospital in China [8] reduced the mean waiting time per month for consultations by 34% (8.1 minutes/23.9 minutes). In addition, 2 public primary care centers in South Africa reported reductions of 21% (27 minutes/129 minutes) and 29% (79 minutes/275 minutes), respectively, in waiting time [70]. This study was also implemented in 3 phases over 8 months, which can explain the higher reduction compared with our study.

In general, we found that our reduction rate falls within the range reported in other published studies using similar methodologies. A long implementation time and the opportunity to incorporate all the components of the intervention could have improved our results.

#### Association Between Patient Satisfaction and Waiting Time

In this study, we found little evidence of association between waiting time and patient satisfaction (OR 1, 95% CI 0.99-1.00; *P*=.37). Another study from China, with a similarly small sample size (49 patients), also reported nonsignificant negative association between time spent at the clinic and satisfaction (*r*=−0.07) [10].

A study at the Hamilton Regional Eye Institute in Canada reported significant association between waiting time and patient satisfaction (OR 0.92, 95% CI 0.86-0.98; *P*=.01) [71]. The study was based on an appointment system and implemented over 8 months, which is more likely to be a sufficient period to explore this relationship. A comparative study between primary care centers in Gauteng and Free State in South Africa found a negative association between patients’ impression about time spent at the clinic and satisfaction [72]. Strong negative association between patient satisfaction and waiting time was also reported among 1403 antenatal care visits in Kenya and 859 in Namibia, across 564 and 303 health facilities, respectively [73]. Negative association was also observed among 1617 patients with HIV, undergoing antiretroviral therapy in Nigeria [74]. In Malawi, negative association between waiting time and patient satisfaction was reported among 120 women undergoing cervical cancer screening, as was the case among 406 participants seeking laboratory services at antiretroviral therapy clinics in Addis Ababa in Ethiopia [75,76].

We report that patients who were dissatisfied commonly complained of long waiting times (13/17, 76%). Other studies from Canada, India, and Cameroon reported similar findings (79%, 73.3%, and 73%, respectively) [77-79].

The decrease in waiting time achieved through our intervention was not reflected in a significant improvement in patient satisfaction after the intervention. We believe that the effect size was not sufficiently large to affect patient satisfaction over a short time at the clinic, and it is possible that a large significant impact on waiting time reduction and a large sample size are needed for it to be a good predictor of patient satisfaction.

### Strengths and Limitations

This is the first quality improvement study in Cameroon with the primary end point of improving waiting time, using a mobile data collection kit for real-time patient monitoring. In addition to providing some evidence in circumstances under which randomized controlled trials may not be possible [80], this study prioritizes and places users at the forefront of quality improvement [81]. The data collection method was automated, thereby reducing data entry errors.

Being the first quality improvement intervention, the change process was slower than expected. The limited influence over contextual factors could have affected the intervention’s degree of success. In addition, not all changes that were proposed were implemented, which also limited the impact of the intervention. The sample size was limited by the data collection method. Each data collector could follow up only a single patient at a time from start to finish. This limited the daily enrollment to a maximum of 2 patients per data collector and sometimes just a single participant, depending on the consultation cycle. A large sample size would have led to a more normally distributed outcome variable and better inference. Finally, we did not perform subgroup analysis of the changes implemented, to measure the impact of each change on waiting time and satisfaction.

The unexpected increase in the waiting time for the general ophthalmologist examination may have been caused by a fast service rate of the preceding units, indicating the importance of considering the patient pathway in its entirety when designing interventions. It was also found that women experienced slight increase in their waiting time. Investigating the reasons for this finding is beyond the scope of this study and would require further exploration in a study with a large sample size.

### Public Health Implications

This study sets the pace for further considerations regarding the delivery of evidence-based patient-centered eye care [82]. There is an urgent need to rethink the eye care delivery strategy in Cameroon [18,83]. The postpandemic era will need even more efficient health systems. This will require patients to be considered as partners in quality improvement. Our intervention is a demonstration of how relatively small investments can lead to service improvements. Further studies are needed to improve waiting time and reduce the opportunity cost of consultation for patients.

### Conclusions

We sought to improve waiting time and patients’ satisfaction using PDSA-led quality improvement. We found 15% borderline significant improvement in waiting time over 6 weeks, suggesting that PDSA-led quality improvement at MICEI is promising over a long period. Our results suggest that improving the waiting time in the short run will not produce significant improvements in patient satisfaction in the setting under study. This study highlights the importance of patient-centered quality improvement, which helps to improve the provider-user relationship. Given the lack of evidence on the acceptable waiting time for a comprehensive eye examination at MICEI, our results provide a benchmark for standardizing the cycle time for a comprehensive eye examination.

We recommend that strategies aimed to reduce waiting time focus on reducing the idling time rather than affecting the consultation time. These may include reducing the number of service points that the patient has to navigate in the clinic and considering placing the consultation with the physician earlier in the patient flow. In addition, introduce a phasic appointment system, starting with reviews and progressively introducing them to new patients. Specific measures introduced with this intervention should be incorporated routinely in the clinic, such as the following: (1) automated patient flow monitoring system that tracks the start and finish times at each service point, (2) introduction of a second receptionist for the separate handling of reviews, (3) implementation of robust ticketing at the gate and reception for all patients, (4) queue length checks along the patient pathway and waiting time threshold alert system for on-the-spot handling of bottlenecks, (5) triaging of patient files led by assistant nurses at the general ophthalmology department, (6) proactive sorting of patient files in the medical records, and (7) use of audiovisual materials for patient education on the patient pathway and waiting time.

## Appendix

Multimedia Appendix 1 Evaluation of contextual factors.

Multimedia Appendix 2 Survey questionnaire.

Multimedia Appendix 3 Distribution of waiting time.

## Abbreviations

MICEI: Magrabi International Council of Ophthalmology Cameroon Eye Institute
ODK: Open Data Kit
OR: odds ratio
PDSA: Plan-Do-Study-Act

## Data Availability

The data set for this study will be made available upon reasonable request. Kindly contact the corresponding author with detailed study protocol and justification of funding to conduct the study.
