# Supplementary material for: Waiting Time and Patient Satisfaction in a Subspecialty Eye Hospital Using a Mobile Data Collection Kit: Pre-Post Quality Improvement Intervention
Source: JMIRx Med. 2022 Aug 9;3(3):e34263. doi: 10.2196/34263 (PMC10414230; doi:10.2196/34263)
Supplement: Multimedia Appendix 1 [file xmed_v3i3e34263_app1.docx]

## Waiting Time and Patient Satisfaction in a Subspecialty Eye Hospital in Cameroon Using a Mobile Data Collection Kit: Pre and Post Quality Improvement Intervention

Table S1: Contextual factors assessment with Model for Understanding Success in Quality

|  | **Totally Neither Totally Don’t**  **agree agree nor disagree know**  **disagree N/A**  **7 6 5 4 3 2 1 0** | | |
| --- | --- | --- | --- |
|  | **Item** | | **Score** |
|  |  | | **132.9** |
|  |  | |  |
| **Contextual factor** | | | |
|  | External motivators | | 5 |
|  | External project sponsorship | | 6 |
|  | Organizational quality improvement leadership | | 6 |
|  | Organization senior leader sponsor | | 6 |
|  | Organization quality improvement culture | | 6 |
|  | Organization quality improvement maturity | | 7 |
|  | Quality improvement workforce focus | | 4.7 |
|  | Resource availability | | 6 |
|  | Data infrastructure | | 5 |
|  | Quality improvement team leadership | | 7 |
|  | Quality improvement team diversity | | 7 |
|  | Quality improvement team subject matter expert | | 5 |
|  | Quality improvement team decision-making processes | | 5.7 |
|  | Quality improvement team norms | | 5.5 |
|  | Team quality improvement skills | | 2 |
|  | Quality improvement team physician involvement | | 7 |
|  | Team prior quality improvement experience | | 3 |
|  | Quality improvement team tenure | | 7 |
|  | Microsystem quality improvement leadership | | 7 |
|  | Microsystem motivation | | 5 |
|  | Microsystem quality improvement capability | | 1 |
|  | Microsystem quality improvement culture | | 5 |
|  | Task strategic importance to the organization | | 7 |
|  | Triggering event | | 7 |
| **Total score guide** | | | |
|  | 168 | Highest possible Model for Understanding Success in Quality score | |
|  | 120-168 | The project has a reasonable chance of success | |
|  | 80-119 | The project could be successful, but possible contextual barriers | |
|  | 50-79 | The project has serious contextual issues and is not set up for success | |
|  | 25-49 | The project should not continue; consider deploying resources to other improvement activities | |
|  | 24 | Lowest possible Model for Understanding Success in Quality score | |
