# Supplementary material for: Waiting Time and Patient Satisfaction in a Subspecialty Eye Hospital Using a Mobile Data Collection Kit: Pre-Post Quality Improvement Intervention
Source: JMIRx Med. 2022 Aug 9;3(3):e34263. doi: 10.2196/34263 (PMC10414230; doi:10.2196/34263)
Supplement: Multimedia Appendix 2 [file xmed_v3i3e34263_app2.pdf]

Waiting Time and Patient Satisfaction in a Subspecialty Eye Hospital in Cameroon Using a Mobile Data Collection Kit: Pre and Post Quality Improvement Intervention

Enumeration Details

1. Interviewer ID:

____

2. Study ID:

____

3. Study date:

yyyy-mm-dd

4. Participant ID:

____

Time Motion

1. At what time did patient arrive?
(verify ticket time at the gate)

hh:mm

2. At what time did patient consent to participate?

hh:mm

3. How many patients are in the waiting area while patient is waiting?

____

4. At what time was patient called at the Reception?

hh:mm

5. At what time did patient finish at the Reception?

hh:mm

6. How was patient classified at the Medical Records?

- ◯ New (1)
- ◯ Review (2)

7. How many patients are waiting to be served at the Medical Records?

____

8. At what time was patient called at the Medical Records?

hh:mm

9. A what time did patient leave the Medical Records?

hh:mm

10. Has patient been sent to the General Ophthalmology?

- ◯ Yes
- ◯ No

11. If Yes, how many patients are waiting in the waiting area?

____

12. At what time was patient called into the first room?

hh:mm

13. Which room was patient called into?

- ◯ Room1 (VA)
- ◯ Room2 (IOP)
- ◯ Room3 (Prescreen)
- ◯ Room4 (Exam)
- ◯ Room5 (refraction)
- ◯ Major (Prescreen)

14. At what time did patient exit from the first room?

hh:mm

15. Was patient called into a sencond room at the General Ophthalmology?

- Yes
- No

16. If Yes, Which room was patient called into?

- Room1 (VA)
- Room2 (IOP)
- Room3 (Prescreen)
- Room4 (Exam)
- Room5 (refraction)
- Major (Prescreen)

17. At what time was patient called into the second room?

hh:mm

18. How many patients are in the waiting area

19. At what time did patient finish in the second

hh:mm

20. Has patient been called into a third room at the General Ophthalmology?

- Yes
- No

21. If Yes, Into which room was patient called?

- Room1 (VA)
- Room2 (IOP)
- Room3 (Prescreen)
- Room4 (Exam)
- Room5 (refraction)
- Major (Prescreen)

22. At what time was patient called into the third room at the General Ophthalmology?

hh:mm

23. How many patients are there in the waiting area?

____

24. At what time did patient exit the third room?

hh:mm

25. Was patient sent into a fourth room at the General Ophthalmology?

- ◯ Yes
- ◯ No

26. If Yes, into which room has patient been called?

- ◯ Room1 (VA)
- ◯ Room2 (IOP)
- ◯ Room3 (Prescreen)
- ◯ Room4 (Exam)
- ◯ Room5 (refraction)
- ◯ Major (Prescreen)

27. At what time did patient enter the fourth room at the General Ophthalmology?

hh:mm

28. How many patients are waiting in the waiting area?

____

29. At what time did patient exit from the fourth room at the General Ophthalmology?

hh:mm

30. Which service was patient sent to?

- ◯ General Ophthalmology (0)
- ◯ Cataract & Glaucoma (1)
- ◯ Retina Unit (2)
- ◯ Cornea & Refractive Errors (3)
- ◯ Paediatric Services (4)
- ◯ Optical Shop (5)
- ◯ Pharmacy (6)
- ◯ Other (7)

Other (specify)

31. Was patient sent to do a fifth exam? At the General Ophthalmology?

- Yes
- No

32. If Yes, which room was patient called into?

- Room1 (VA)
- Room2 (IOP)
- Room3 (Prescreen)
- Room4 (Exam)
- Room5 (refraction)
- Major (Prescreen)

33. At what time was patient called to do the fifth exam?

hh:mm

34. How many patients are currently waiting in the waiting area?

35. At what time did patient finish in the fifth room?

hh:mm

36. Which service was patient sent to?

- Cataract & Glaucoma (1)
- Retina Unit (2)
- Cornea & Refractive Errors (3)
- Paediatric Services (4)
- Optical Shop (5)
- Pharmacy (6)
- Other (7)

Other (specify)

37. What patient sent to the Cataract and Glaucoma unit?

- Yes
- No

38. If Yes, How many patients are currently waiting to be called at this service?

____

39. At what time was patient called to do the first exam?

hh:mm

40. Which room has patient been called into?

- ○ Room 6 & 7
- ○ Room 8 & 9
- ○ Room 10
- ○ Room 11
- ○ Room 12
- ○ Room 13
- ○ Room 14
- ○ Room 15

41. At what time did patient finish with the first exam?

hh:mm

42. Has patient been called for a second exam at this unit?

- ○ Yes
- ○ No

43. How many persons are waiting to be called at this unit.

____

44. At what time was patient called to do the second exam?

hh:mm

45. Which room has patient been called into?

- Room 6 & 7
- Room 8 & 9
- Room 10
- Room 11
- Room 12
- Room 13
- Room 14
- Room 15

46. At what time did the patient finish the second exam?

hh:mm

47. Is the patient invited to do a third exam at this unit?

- Yes
- No

48. If Yes, At what time was patient called for the third exam?

hh:mm

49. In which room has patient been called?

- Room 6 & 7
- Room 8 & 9
- Room 10
- Room 11
- Room 12
- Room 13
- Room 14
- Room 15

50. At what time did patient finish with the third exam?

hh:mm

51. Has the patient been sent to Room 4 of the General ophthalmology?

- Yes
- No

52. If Yes, at what time did the patient enter into Room 4?

hh:mm

53. At what time did the patient exit either from Room 4?

hh:mm

54. Which service was patient sent to?

- ◯ Cataract & Glaucoma (1)
- ◯ Retina Unit (2)
- ◯ Cornea & Refractive Errors (3)
- ◯ Paediatric Services (4)
- ◯ Optical Shop (5)
- ◯ Pharmacy (6)
- ◯ Other (7)

Other (please specify)

55. Has the patient been sent to the Retina unit?

- ◯ Yes
- ◯ No

56. If Yes, how many patients are waiting to be seen at this unit?

57. At what time was patient called for the first exam in this unit?

hh:mm

58. Which was patient called into?

- ◯ Room 6 & 7
- ◯ Room 8 & 9
- ◯ Room 10
- ◯ Room 11
- ◯ Room 12
- ◯ Room 13
- ◯ Room 14
- ◯ Room 15

59. At what time did patient finish his/her first exam?

hh:mm

60. At what time was patient called for the second exam?

hh:mm

61. Into which room was patient called for the second exam?

- Room 6 & 7
- Room 8 & 9
- Room 10
- Room 11
- Room 12
- Room 13
- Room 14
- Room 15

62. At what time did patient finish his/her second exam?

hh:mm

63. Was patient called to do a third exam at this unit?

- Yes
- No

64. If Yes, how many patients are waiting to be served at this unit?

65. At what time was called for the third exam at this unit?

hh:mm

66. Which room was patient called into?

- Room 6 & 7
- Room 8 & 9
- Room 10
- Room 11
- Room 12
- Room 13
- Room 14
- Room 15

67. At what time did patient finish with the third exam?

hh:mm

68. Has the patient been sent to another room?

- Yes
- No

69. If Yes, which room was patient called into?

- Room4 (Oph)
- Room 6 & 7
- Room 13
- Room 14
- Room 15

70. How many patients are waiting in the waiting area?

71. At what time was patient called into the indicated room?

hh:mm

72. At what time did patient exit from the room?

hh:mm

73. Which service/unit is the patient sent?

- Cataract & Glaucoma (1)
- Retina Unit (2)
- Cornea & Refractive Errors (3)
- Paediatric Services (4)
- Optical Shop (5)
- Pharmacy (6)
- Other (7)

Other (please specify)

74. Has patient been sent to the Cornea and Refractive Errors unit?

- Yes
- No

75. If Yes, how many patients are waiting to be served in this unit?

76. Has patient been called into Room 18 for refraction?

- Yes
- No

77. At what time was patient called into Room 18?

hh:mm

78. At what time did patient finish with the refraction?

hh:mm

79. Has patient been called into Room 16 to see the doctor?

- Yes
- No

80. If Yes, at what time was patient called into Room 16?

hh:mm

81. How many patients are waiting into be seen in this unit?

82. At what time did patient finish seeing the doctor in Room 16?

hh:mm

83. Was patient called do cornea topography in Rooms 22-23

- Yes
- No

84. If Yes, at what time was patient called into Rooms 22-23?

hh:mm

85. At what time did patient exit from Rooms 22-23?

hh:mm

86. Has patient been called to see the second ophthalmologist?

- Yes
- No

87. If Yes, at what time did patient finish with the second Ophthalmologist?

hh:mm

88. Has patient been sent to another room or service?

- Yes
- No

89. If Yes, which room was patient sent to?

- Room 6 & 7
- Room 8 & 9
- Room 4
- Room 10
- Room 13
- Room 14
- Room 15

90. How many patients are waiting in the waiting area?

91. At what time did patient enter into the indicated room?

hh:mm

92. At what time did patient exit from the room?

hh:mm

93. Which service/unit was patient sent to?

- Cataract & Glaucoma (1)
- Retina Unit (2)
- Cornea & Refractive Errors (3)
- Paediatric Services (4)
- Optical Shop (5)
- Pharmacy (6)
- Other (7)

Other (please specify)

94. Le patient est-il envoyé au Pédiatrie? Was patient sent to the Pediatric unit

- Yes
- No

95. If Yes, how many patients are waiting to see Doctor?

96. At what time was patient called to see the Doctor?

hh:mm

97. At what time did patient finish with the Doctor

hh:mm

98. Has patient been sent to another service unit?

- Yes
- No

99. If Yes, which room was patient sent to?

- Room 6 & 7
- Room 8 & 9
- Room 4
- Room 10
- Room 13
- Room 14
- Room 15
- Room 16
- Room 22 & 23

100. How many patients are waiting at this service unit?

____

101. At what time was patient called into the room?

hh:mm

102. At what time did patient exit from the room?

hh:mm

103. Which service was patient sent to?

- Cataract & Glaucoma (1)
- Retina Unit (2)
- Cornea & Refractive Errors (3)
- Paediatric Services (4)
- Optical Shop (5)
- Pharmacy (6)
- Other (7)

Other (please specify)

____

104. What is the main condition patient has been diagnosed with?

____

105. Has patient been sent to the Optical Shop?

- Yes
- No

106. If Yes, how many patients are waiting to be served?

____

107. At what time was patient received at the Optical Shop?

hh:mm

108. At what time did patient finish with the Optical Shop?

hh:mm

109. Has the patient been sent to the Pharmacy?

- Yes
- No

110. If Yes, how many patients are waiting to be seen at the Pharmacy

____

111. At what time was patient called up at the Pharmacy?

hh:mm

112. At what time did patient finish with the Pharmacy?

hh:mm

113. Has patient been sent to or did he/she visit the Optical Shop?

- Yes
- No

114. If Yes, how many persons are waiting at the Optical Shop

____

115. At what time was patient received at the Optical Shop?

hh:mm

116. At what time did patient finish with the Optical Shop?

hh:mm

117. Has patient been sent to the Admissions Office?

- Yes
- No

118. If Yes, how many patients are waiting at the Admissions

____

119 At what time was patient recieved at the Admissions Office?

hh:mm

120. At what time did patient finish with the Admissions Office?

hh:mm

121. Has patient been sent to the Pharmacy?

- Yes
- No

122. If Yes, how many patients are waiting at the Pharmacy?

____

123. At what time was patient received at the Pharmacy?

hh:mm

124. At what time did patient finish at the Pharmacy?

hh:mm

125. Has patient been sent to the Laboratory?

- Yes
- No

126. If Yes, How many patients are waiting to see the Laboratory Technician?

____

127. At what time was patient called to see the Laboratory Technician?

hh:mm

128. At what time did patient finish with the Laboratory Technician?

hh:mm

129. Has patient been sent to Rooms 6 & 7 for preoperative tests

- Yes
- No

130. If Yes, how many patients are waiting to be served?

131. At what time was patient called into Rooms 6 & 7 for bieometry and B scan?

hh:mm

132. At what time did patient finish wiht preoperative tests? In Rooms 6 & 7

hh:mm

133. At what time did patient finish with the consultation at the clinic?

hh:mm

Socio-Demographic Variables

1. Participant Year of Birth
Enter year of birth (Not age)

2. Sex of Participant

- Male (1)
- Female (2)
- Prefer not to say (3)

3. Participant's marital status

- Married (1)
- Cohabiting (2)
- Single (3)
- Divorced/Widow (4)

4. Participant's region of origin

- Littoral (1)
- Extreme Nord (2)
- Adamawa (3)
- Centre (4)
- West (5)
- North West (6)
- East (7)
- North (8)
- South (9)
- South West (10)
- Other

Other (please specify)

5. Participant's region of residence

- Littoral (1)
- Extreme Nord (2)
- Adamawa (3)
- Centre (4)
- West (5)
- North West (6)
- East (7)
- North (8)
- South (9)
- South West (10)
- Other

Other (please specify)

Socio-Economic Variables

1. Who is responding?

- ◯ Participant (1)
- ◯ Proxy (2)

2. Has your household got electricity?

- ◯ Yes
- ◯ No
- ◯ Don't Know (99)

3. Has your household got a television set?

- ◯ Yes
- ◯ No
- ◯ Don't Know (99)

4. Has your household got a referigerator?

- ◯ Yes
- ◯ No
- ◯ Don't Know (99)

5. Has your household got a CD/DVD player?

- ◯ Yes
- ◯ No
- ◯ Don't Know (99)

6. Has your household got a ward robe?

- ◯ Yes
- ◯ No
- ◯ Don't Know (99)

7. Has your household got a generator or solar panel?

- ◯ Yes
- ◯ No
- ◯ Don't Know (99)

8. Do you own either a bicycle or motorbike?

- ◯ Yes
- ◯ No
- ◯ Don't Know (99)

9. Do you own a wrist watch?

- ◯ Yes
- ◯ No
- ◯ Don't Know (99)

10. Do you own a mobile telephone

- ◯ Yes
- ◯ No
- ◯ Don't Know (99)

11. Do you own a bank account?

- ◯ Yes
- ◯ No
- ◯ Don't Know (99)

12. Do you have a mobile money account?

- ◯ Yes
- ◯ No
- ◯ Don't Know (99)

13. What is the main source of protable water for your household during the rainy season?

- ◯ Piped into dwelling (1)
- ◯ Personal modern well water (2)
- ◯ Buy from neighbour (3)
- ◯ Carry from bush spring (4)
- ◯ Other (5)

Other (please specify)

____

14. What is the main material for the walls of your house

- ◯ Ceramic Tiles (1)
- ◯ Cement Blocks (2)
- ◯ Ground bricks (3)
- ◯ Wood planks (4)
- ◯ Palm/bamboo/thatch (5)
- ◯ Other (6)

Other (please specify)

---

15. What type of fuel is used for cooking in your household?

- ◯ Gas (1)
- ◯ Charcoal (2)
- ◯ Wood (3)
- ◯ Other (4)

Other (please specify)

---

16. What transport means did you use to the clinic?

- ◯ Private transport (1)
- ◯ Public transport (2)
- ◯ Motorbike (3)
- ◯ Other (4)

Other (specify)

---

17. What is the principal material for the floor in your house?

- ◯ Ceramic tiles (1)
- ◯ Cement (2)
- ◯ Ground (3)
- ◯ Wood planks (4)
- ◯ Other (5)

Other (please specify)

---

18. What toilet facility is used by your household?

- ◯ Personal flushing toilet (1)
- ◯ Shared flushing toilet (2)
- ◯ Pit latrine (3)
- ◯ Other (4)

Other (please specify)

---

19. How long did it take to get to the clinic?

- < 1 hour (1)
- A few hours (2)
- Half a day (3)
- 1 day (4)
- 1-2 days (5)
- 2-3 days (6)
- Other (7)

Other (please specify)

____

Occupation

1. Have you worked during the last 7 days?

- Yes
- No

2. Do you have a job or enterprise from which you were absent due to leave, sickness, holidays or any other reason?

- Yes
- No

3. Have you worked during the last 12 months

- Yes
- No

4. What have you been doing for most of the time during the last 12 months?

- Going to school/studying (1)
- Looking for work (2)
- Retired (3)
- Doing farming (4)
- Too ill to work (5)
- Physically unable to work (6)
- Housework/childcare (7)
- Other (8)

Other (please specify)

____

5. What type of work do you usually do?

____

Education

1. What level of education have you attained?

- None (0)
- Primary (1)
- Secondary (O/L) (2)
- Secondary (A/L) (3)
- University (first degree) (4)
- Post first degree (5)

Visit to Other Facilities

1. Have you been to a herbalist (traditional doctor) for eye problems?

- Yes
- No

2. Have you been to a public hospital for eye problems?

- Yes
- No

3. Have your been to a private clinic for eye problems?

- Yes
- No

4. Have you been to a pharmacy for eye problems?

- Yes
- No

5. Have you visited an optical shop or optician for eye problems?

- Yes
- No

Patient Awareness (Sensibilization)

1. How did you learn about our hospital?

- Insurance/ company (1)
- Friend/Family (2)
- Community health worker (3)
- Former patient (4)
- Other (5)

Other (please specify)

Past Satisfaction (Satisfaction Antérieure)

1. Is this your first visit to this hospital?

Assurez vous que c'est un Ancien Patient

- Yes
- No

2. If No, were you satisfied during your last visit?

- Yes
- No

Current Satisfaction (Satisfaction Actuelle)

1. Satisfied with time to wait for services

- Yes
- No

2. If No, what happened?

____

3. Would you like to share any other comments in regards to any staff or services during this visit?

____
