# Supplementary material for: Waiting Time and Patient Satisfaction in a Subspecialty Eye Hospital Using a Mobile Data Collection Kit: Pre-Post Quality Improvement Intervention
Source: JMIRx Med. 2022 Aug 9;3(3):e34263. doi: 10.2196/34263 (PMC10414230; doi:10.2196/34263)
Supplement: Multimedia Appendix 3 [file xmed_v3i3e34263_app3.docx]

## Waiting Time and Patient Satisfaction in a Subspecialty Eye Hospital in Cameroon Using a Mobile Data Collection Kit: Pre and Post Quality Improvement Intervention

**Table S1: Waiting time, service time, and idling time (minutes) by gender**

|  | | Pre-intervention | | | | | | Post-intervention | | | | | |
| --- | --- | --- | --- | --- | --- | --- | --- | --- | --- | --- | --- | --- | --- |
|  | | Men | | | Women | | | Men | | | Women | | |
| Service Point | | Wait time | Service time | Idling time | Wait time | Service time | Idling time | Wait time | Service time | Idling time | Wait time | Service time | Idling time |
|  | | Mean (SD) | Mean (SD) | Mean (SD) | Mean (SD) | Mean (SD) | Mean (SD) | Mean (SD) | Mean (SD) | Mean (SD) | Mean (SD) | Mean (SD) | Mean (SD) |
|  |  |  |  |  |  |  |  |  |  |  |  |  |  |
|  |  | **472.1 (86.5)** | **110.3 (45.3)** | **361.8 (84.9)** | **428.7**  **(104.2)** | **113.4**  **(50.4)** | **315.3**  **(107.4)** | **330**  **(104.3)** | **76.8**  **(29.0)** | **253.2**  **(92.9)** | **438.4**  **(141.5)** | **93**  **(31.8)** | **345.4**  **(131.3)** |
|  | Reception | 76.8  (44.2) | 31.1  (37.1) | 45.7  (42.5) | 51.2  (36.7) | 15.8  (16.8) | 35.4  (37.5) | 49.6  (33.5) | 18.3  (17.8) | 31.3  (31.7) | 67.8  (27.1) | 27.5  (16.5) | 40.3  (28.3) |
|  | Medical records | 29.5  (28.2) | 19.1  (27.9) | 10.4  (9.2) | 42.5  (40.4) | 27.4  (26.7) | 15.1  (16.6) | 19.6  (16.1) | 9.9  (8.3) | 9.7  (13.6) | 29.3  (15.2) | 17.8  (12.2) | 11.5  (11.4) |
| General Ophthalmology | | | | | | | | | | | | | |
|  | Room 1 | 28.1  (23.0) | 5.3  (1.8) | 22.8  (22.7) | 63.9  (50.5) | 20.5  (21.8) | 43.4  (34.1) | 26.2  (11.3) | 10.8  (4.4) | 15.3  (12.4) | 26.4  (10.1)) | 9.1  (3.4) | 17.3  (10.7) |
|  | Room 2 | 40.8  (31.0) | 8.4  (6.9) | 32.4  (25.8) | 34.4  (25.2) | 8.4  (3.4) | 26  (22.6) | 48.3  (30.6) | 8.3  (4.8) | 40  (30.4) | 59.2  (63.7) | 7.2  (6.4) | 52  (63.9) |
|  | Room 3 | 71  (78.6) | 6.6  (2.4) | 64.4  (78.5) | 35  (16.3) | 12.3  (9.4) | 22.7  (12.4) | 60  (-) | 10  (-) | 50  (-) | 59  (26.9) | 6  (0) | 53  (26.9) |
|  | Major | 49.7  (31.2) | 10  (10) | 39.7  (31.7) | 85  (-) | 4  (-) | 81  (-) | 58  (-) | 8  (-) | 50  (-) | 37  (4.2) | 11.5  (3.5) | 25.5  (0 .7) |
|  | Room 4 | 43.5  (45.2) | 4.7  (1.5) | 38.8  (45.7) | 91.7  (61.1) | 14.2  (12.5) | 77.5  (51.6) | 68.3  (31.6) | 21.2  (15.5) | 47.1  (30.7) | 140.2  (136.8)) | 15.2  (10.8)) | 125  (130.7) |
|  | Room 5 | - | - | - | - | - | - | 205  (-) | 62  (-) | 143  (-) | 10.5  (2.6) | 3.5  (1) | 7  (3.2) |
| Cataract and Glaucoma | | | | | | | | | | | | | |
|  | Room 6-7 | 20  (-) | 15  (-) | 5  (-) | 30  (0) | 18  (2.8) | 12  (2.8) | - | - | - | - | - | - |
|  | Room 13 | 182.5  (41) | 17  (6.3) | 165.5  (39.3) | 189  (61.7) | 19.3  (7.8) | 169.7  (69.4) | 125  (-) | 19  (-) | 106  (-) | 155.7  (91.2) | 23.7  (3.2) | 132  (93.6) |
|  | Room 14 | - | - | - | - | - | - | - | - | - | 131  (-) | 6  (-) | 125  (-) |
|  | Room 15 | 154.2  (49.8) | 9.5  (4.2) | 144.7  (49.6) | 303  (11.3) | 5.5  (0.7) | 297.5  (12.0) | 120  (48.8) | 11.7  (2.1) | 108.3  (47.5) | 151  (87.3) | 8.6  (1.9) | 142.4  (88.8) |
|  | **Retina** | | | | | | | | | | | | |
|  | Room 12 | 82  (-) | 10  (-) | 72  (-) | 131  (-) | 110  (-) | 21  (-) | 49  (-) | 21  (-) | 28  (-) | - | - | - |
|  | Room 8-9 | - | - | - | - | - | - | - | - | - | 56  (-) | 54  (-) | 2  (-) |
| Cornea and Refractive Errors | | | | | | | | | | | | | |
|  | Room 18 | 133.6  (83.1) | 15.3  (5.0) | 118.3  (81.3) | 188  (31.2) | 26.3  (15.0) | 161.7  (46.2) | 94  (8.6) | 13  (6.8) | 81  (9.7) | 134.2  (63.7) | 13.2  (3.9) | 121  (66.6) |
|  | Room 16 | 197  (74.9) | 18  (4.2) | 179  (79.2) | 33.7  (12.0) | 12.7  (6.8) | 21  (15.5) | 148.3  (81.3) | 14  (5.3) | 134.3  (85.9) | 81.7  (46.5) | 10  (2.6) | 71.7  (49.0) |
| Pediatrics | | | | | | | | | | | | | |
|  | Room 20 | 103  (-) | 8  (-) | 95  (-) | 303  (-) | 13  (-) | 290  (-) | 155  (-) | 12  (-) | 143  (-) | 240  (80.6) | 17  (11.3) | 223  (69.3) |
|  |  |  |  |  |  |  |  |  |  |  |  |  |  |
|  | Optical shop | 38  (29.7) | 25.6  (29.2) | 12.4  (15.7) | 16  (9.6) | 12.6  (9.3) | 3.4  (1.5) | 8.5  (6.4) | 2.5  (0 .7) | 6  (7.1) | 4  (2.2) | 2.2  (1.5) | 1.8  (1.0) |
|  | Pharmacy | 22  (15.3) | 11.9  (7.8) | 10.1  (16.3) | 33.7  (48.3) | 10.8  (5.8) | 22.9  (44.4) | 14.7  (6.0) | 5.2  (2.8) | 9.5  (7.2) | 8.4  (5.4) | 4.4  (2.4) | 4  (3.7) |
|  | Admissions | 31  (-) | 26  (-) | 5  (-) | - | - | - | - | - | - | 20  (-) | 13  (-) | 17  (-) |

**Table S2: Patient flow service point codes**

| Service point code | Code description |
| --- | --- |
| Room 1 | Visual acuity, blood pressure, random blood sugar |
| Room 2 | Visual acuity and intraocular pressure |
| Room 3 | Prescreening (anterior chamber exam) |
| Senior nurse Office (general ophthalmology) | Prescreening (anterior chamber exam) |
| Room 4 | General ophthalmologist (anterior and posterior assessment) |
| Room 5 | Refraction (objective and subjective) |
| Room 6 | Investigations (visual field) |
| Room 7 | Intra-ocular lens, tonometry, pachymetry, and biometry |
| Room 8 | Retina specialist exam |
| Room 9 | Retina specialist exam |
| Room 10 | Lasers |
| Room 11 | Investigation (angiography/fundus photography) |
| Room 12 | Optical Coherence Tomography (OCT) investigation |
| Room 13 | Refraction |
| Room 14 | Refraction |
| Room 15 | Glaucoma specialist exam |
| Room 16 | Cornea and refractive errors ophthalmologist exam |
| Room 17 | Auto refraction |
| Room 18 | Refraction |
| Room 19 | Orthoptist assessment |
| Room 20 | Ophthalmologist exam |
| Room 22 | Corneal topography |
| Room 23 | Wave scan |
